# Interfaces and Oxygen Vacancies-Enriched Catalysts Derived from Cu-Mn-Al Hydrotalcite towards High-Efficient Water–Gas Shift Reaction

**DOI:** 10.3390/molecules28041522

**Published:** 2023-02-04

**Authors:** Hanci Li, Zhenyi Xiao, Pei Liu, Hairu Wang, Jiajun Geng, Huibin Lei, Ou Zhuo

**Affiliations:** 1 College of Chemistry and Chemical Engineering, Jishou University, Jishou 416000, China; 2 Hunan Province Key Laboratory of Mineral Cleaner Production and Green Functional Materials, Jishou University, Jishou 416000, China

**Keywords:** water–gas shift, hydrotalcite, oxygen vacancies, interface, hydrogen

## Abstract

The water–gas shift (WGS) reaction is an important process in the hydrogen industry, and its catalysts are of vital importance for this process. However, it is still a great challenge to develop catalysts with both high activity and high stability. Herein, a series of high-purity Cu-Mn-Al hydrotalcites with high Cu content have been prepared, and the WGS performance of the Cu-Mn-Al catalysts derived from these hydrotalcites have been studied. The results show that the Cu-Mn-Al catalysts have both outstanding catalytic activity and excellent stability. The optimized Cu-Mn-Al catalyst has displayed a superior reaction rate of 42.6 ${\mu \mathrm{mol}}_{\mathrm{CO}}^{-1}\cdot g_{\mathrm{cat}}^{-1}\cdot s^{-1}$, while the CO conversion was as high as 96.1% simultaneously. The outstanding catalytic activities of the Cu-Mn-Al catalysts could be ascribed to the enriched interfaces between Cu-containing particles and manganese oxide particles, and/or abundant oxygen vacancies. The excellent catalytic stability of the Cu-Mn-Al catalysts may be benefitting from the low valence state of the manganese of manganese oxides, because the low valence manganese oxides have good anti-sintering properties and can stabilize oxygen vacancies. This study provides an example for the construction of high-performance catalysts by using two-dimensional hydrotalcite materials as precursors.

## 1. Introduction

Hydrogen gas is not only a kind of vital chemical raw material, but it is also a kind of clean energy carrier with bright prospects. To date, approximately 95% of the appreciable supply is produced from reforming gas, which originates from the reforming of natural gas, coal, biomass, and organic wastes [1,2]. The reforming gas usually contains an appreciable amount of CO, and the water–gas shift (WGS) reaction is used to transfer CO into hydrogen and CO_2_ [3]. Pure hydrogen production is obtained by removing the easily separated CO_2_ and excess H_2_O from the outflow gas of the WGS reaction. A high CO conversion is critically important for the production of pure hydrogen. However, the WGS reaction is a reversible exothermic reaction (CO + H_2_O ⇌ CO_2_ + H_2_, ΔH = −41.1 kJ·mol^−1^) [1,3]. It means that the low reaction temperature favors high CO equilibrium conversion but causes a low reaction rate. Thus, it is significant to develop high active catalysts with high CO conversion for the WGS reaction.

Cu-based catalysts are widely adopted for the low-temperature WGS reaction due to their high catalytic activity and relatively low cost. Since the 1960s, the Cu/ZnO/Al_2_O_3_ catalyst has been used in industrial WGS applications [4]. For decades, many Cu-based catalysts have been prepared and their performance in WGS reactions, such as Cu/CeO_2_ [5,6], Cu/ZnO [7,8], CuO-Fe_2_O_3_/SiO_2_ [9], Cu/MgO/Al_2_O_3_ [10,11], and Cu-Mn spinel oxide, have been deeply investigated [12,13]. Further studies have been carried out to reveal the active site and catalytic mechanism. For Cu/CeO_2_ catalysts, it has been proposed that the active sites of low-temperature WGS reactions are located at the Cu-CeO_2_ interface [5,6,14]. Chen and his co-workers [5] suggested that the Cu^+^ site and the neighboring oxygen vacancies (O_v_) of the ceria site at the interface of Cu/CeO_2_ (Cu^+^-O_v_-Ce^3+^) are the active sites for the WGS reaction: the Cu^+^ site chemically adsorbs CO, whereas the neighboring O_v_-Ce^3+^ site dissociatively activates H_2_O. In Cu/ZnO catalysts, the Cu-hydroxylated ZnO ensemble is considered as the active site [8]. In brief, it is generally believed that the active sites of WGS reactions are closely correlated with the interfaces between Cu species and supports.

Hydrotalcite-type compounds, also known as layered double hydroxides (LDH), are a class of two-dimensional (2D) layered materials, which are widely used as catalysts or catalyst precursors [15,16,17]. A key structural feature of LDH is that the divalent and trivalent metal cations are homogeneously distributed in LDH layers at an atomic level [l4,15]. Benefitting from this feature, the catalysts derived from LDH precursors usually exhibit a high dispersion of the metal particles and abundant interfaces between the metal particles and oxide supports [15]. The Cu/ZnO/Al_2_O_3_ catalysts, prepared by calcining and activating the Cu-Zn-Al LDH, have been studied for the catalytic performance of the WGS reaction in 1995 [18]. Afterwards, by improving preparation method, optimizing composition or adding promoter in the Cu-Zn-Al LDH, various Cu/ZnO/Al_2_O_3_ catalysts were developed with excellent performance [4,19,20,21]. The Cu/MgO/Al_2_O_3_ catalysts derived from Cu-Mg-Al LDH also displayed outstanding catalytic performance [10,11,22]. In addition, Cu-Al LDH [11], Ni-Al LDH [23], Ni-Ti LDH [24], and Ni-Cr LDH [25] are used as precursors for WGS catalysts. These reports believe that the excellent catalytic activities of these catalysts are benefitting from the well dispersion of active phase and/or abundant interfaces of metal-support. However, the catalytic stability of these catalysts for the WGS reaction is not satisfactory, and strategies for improving the stability were seldom discussed.

Creating oxygen vacancies is an effective approach to enhance the activation of H_2_O, and, therefore, improve the catalytic activities of WGS catalysts [5,6,26]. It is well known that adding manganese components into the solid catalysts can make oxygen vacancies [27,28,29]. Catalysts derived from Cu-Mn-Al LDH are rich with oxygen vacancies and exhibit good catalytic performances in hydrogenation and oxidation reactions [30,31]. However, using Cu-Mn-Al LDH as the catalyst precursor is still absent for the WGS reaction. In this study, a series of high-purity Cu-Mn-Al LDH with high Cu contents have been prepared by the coprecipitation method under low supersaturation. The catalytic performance of the Cu-Mn-Al catalysts derived from these LDH samples have been studied in WGS conditions, and the underlining structure–function relationships have been discussed.

## 2. Experimental Section

### 2.1. Preparation of Hydrotalcite Samples

All hydrotalcite samples are prepared by the coprecipitation method. In a typical procedure, solution A (100 mL) was prepared by dissolving a mixture of metal nitrates (Cu(NO_3_)_2_, Mn(NO_3_)_2_, and Al(NO_3_)_3_ with a total amount of 0.06 mol) in deionized water. Solution B (100 mL) was obtained by dissolving 0.12 mol of NaOH in deionized water. Solution A and B were simultaneously pumped into a three-neck flask containing 100 mL of Na_2_CO_3_ solution by two peristaltic pumps. To keep hydroxyl ions at a low supersaturation, the flow rates of solution A and solution B were controlled equally at 5 mL/min. The slurry was stirred slowly at 60 °C for 12 h. Then, the resulting precipitate was filtered, washed with deionized water, and dried at 80 °C for 10 h. The solid sample was ground to fine powders and labelled as Cu*_x_*Mn*_y_*Al*_z_*-LDH, where *x*, *y*, and *z* are the designed molar percentage of corresponding metal in the total metal amounts. The obtained sample was heated in air from atmospheric temperature to 773 K by 10 K·min^−1^ and kept for 3 h. The calcined sample was denoted as Cu*_x_*Mn*_y_*Al*_z_*-MMO and was ready for use. For comparison, the Cu-Zn-Al LDH and Cu-Mg-Al LDH were also prepared with *x* = 50, *y* = 25, and *z* = 25. The CuMn sample is also prepared by the same method with *x* = 50 and *y* = 50.

### 2.2. Catalyst Characterization

X-ray diffraction (XRD) patterns were collected on a TD-3500 X-ray diffractometer (Dandong Tongda Instrument Co., Dandong, China) with a Cu *K*_a_ source (k = 0.154 nm) at 40 kV and 30 mA. The metal content of the samples was determined by inductively coupled plasma optical emission spectroscopy (ICP-OES, PerkinElmer Avio 200). N_2_ adsorption/desorption isotherm was measured on a surface area and pore size analyzer (Quantachrome Nova 2000e). All samples were outgassed prior to analysis at 200 °C for 12 h. Specific surface area (*S*_BET_) was calculated via the multipoint BET method, and pore size distributions were calculated by using the non-local density functional theory (NLDFT) equilibrium model (N_2_ at 77 K, cylindr. pore on silica). Transmission electron microscopy (TEM) and high-resolution TEM (HRTEM) were carried out on a Talos F200S instrument operated at an accelerating voltage of 200 kV. The high angle annular dark-field (HAADF) image and the corresponding energy dispersive X-ray spectroscopy (EDX) mappings were recorded on a SUPER X detector. X-ray photoelectron spectroscopy (XPS) was measured through a Thermo Scientific K-Alpha XPS spectrometer. Binding energies were calibrated based on the graphite C1s peak at 284.8 eV. The electron paramagnetic resonance (EPR) of the solid samples was determined at room temperature on an EMX Plus EPR spectrometer (Bruker BioSpin). Before the XPS and EPR measures, the catalyst samples were first reduced for 0.5 h in a H_2_ atmosphere at 300 °C and, then, operated in a WGS condition for 5 h.

### 2.3. Catalytic Testing

The WGS reaction tests were performed on a fixed bed reactor with a diameter of 8 mm. The reaction temperature was automatically controlled by a PID temperature controller with a thermocouple inserted into the center of the catalyst bed. Typically, 50 mg of catalyst and 3.0 g of quartz sand (40–80 mesh) were mixed evenly and charged into the reactor. The catalyst was reduced with pure H_2_ (20 mL·min^−1^) for 0.5 h at 300 °C. After cooling to 200 °C, a gas mixture of CO/H_2_/CO_2_/N_2_ (molar ratio: 14.9/27.2/7.3/50.6) was fed into the reactor with flow rate of 20 mL·min^−1^, corresponding to the gas hourly space velocity (GHSV) of 24,000 mL·g^−1^·h^−1^. The deionized water was injected into the gasification chamber by a quantitative pump, and the produced steam fully mixed with the gas flow. The tests were operated at an elevated temperature from 200 to 400 °C. The outlet gas was analyzed by a gas chromatography system (Qiyang GC9860) equipped with a thermal conductivity detector and a flame ionization detector.

## 3. Results and Discussion

**Figure 1:**
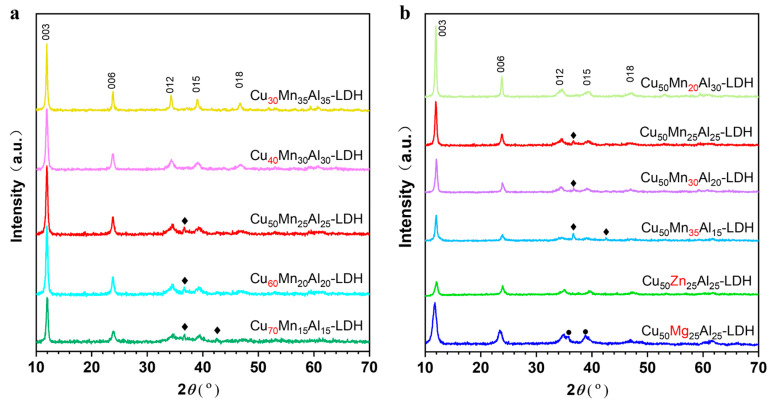
XRD patterns of the LDH precursors. (**a**) Cu*_x_*Mn*_y_*Al*_z_*-LDH. (**b**) Cu*_x_*Mg*_y_*Al*_z_*-LDH, Cu*_x_*Zn*_y_*Al*_z_*-LDH and Cu_50_Mn*_y_*Al*_z_*-LDH. The diffraction peaks marked by the symbols (♦, ●) and are corresponding to the Cu_2_O phase (♦ JCPDS No. 05-0667) and CuO phase (● JCPDS No. 45-0937).

Figure 1 shows the XRD patterns of the LDH samples. The (003), (006), (012), (015) and (018) diffraction peaks are attributed to hydrotalcite-like materials (JCPDS No. 37-0630). Apart from these peaks, there are only a few extremely weak diffraction peaks. It suggests that all the samples are of an almost pure LDH phase. As known, due to the Jahn-Teller effect of copper ions, it is a challenge to prepare pure Cu-containing LDH phases, especially with high copper contents. In this study, by keeping hydroxyl ions under low supersaturation in the preparation process, the by-phases have been successfully suppressed at a low level for all the LDH samples. Curiously, the by-phase usually is CuO in Cu-Mg-Al-LDH and Cu-Zn-Al-LDH samples (Figure 1b) [32,33], but it is cuprite phase (Cu_2_O, JCPDS No. 05-0667) in Cu-Mn-Al LDH samples (Figure 1). This phenomenon has also been observed in previous research [34]. After calcination, the LDH phases disappeared and transformed to corresponding mixed metal oxides (MMO) (Figure S1 in Supplementary Materials). XRD patterns of the MMO samples were only present the diffraction peaks of MnAl_2_O_4_ and CuO phases, and no diffraction peak was related to manganese-containing oxide or other phase (Figure S1). In addition, the XRD pattern shows that the CuMn sample mainly contained CuO and CuMn_2_O_4_ phases (Figure S2).

Table 1 shows the designed contents of metals (the designed percentage of metals in the preparation process), the detected contents of metals, and the specific surface area (*S*_BET_) for all samples. The detected metal contents of the samples are very close to the corresponding designed contents (no more than 1.2 at.% deviation), except for the Al content of the Cu_50_Mg_25_Al_25_-MMO sample. Such precise control of metal contents provides favorable conditions for the comparative studies. In the following discussions, the metal contents of the samples will be expressed using the designed percentage. For the MMO samples at a fixed Mn/Al ratio of 1/1, the *S*_BET_ decreases from 122 to 71 m^2^·g^−1^ as the Cu content increases from 30 to 70%. For the MMO samples at a fixed Cu content of 50%, the *S*_BET_ increases from 72 to 88 m^2^·g^−1^, with the Mn content increasing from 20 to 25%, and decreases to 62 m^2^·g^−1^ when the Mn content reaches 35%. The *S*_BET_ of the Cu_50_Zn_25_Al_25_-MMO and Cu_50_Mg_25_Al_25_-MMO samples were only 67 and 58 m^2^·g^−1^, respectively, evidently lower than that of the Cu_50_Mn_25_Al_25_-MMO sample (88 m^2^·g^−1^). These results suggest that the addition of manganese into LDH can increase the *S*_BET_ of the derived MMO.

**Figure 2:**
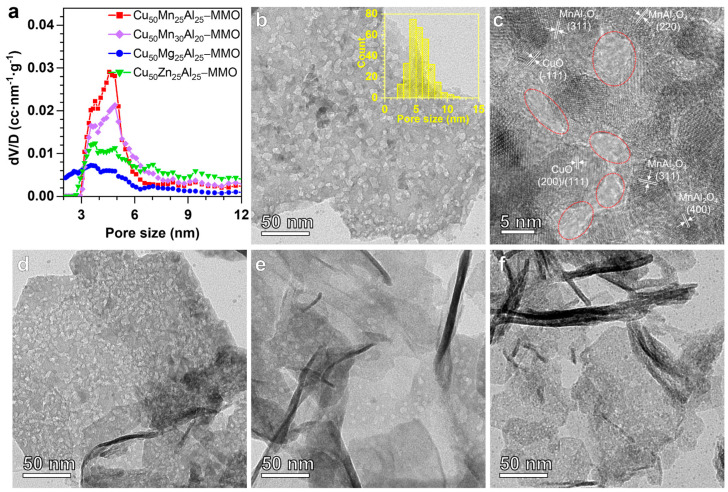
Pore size distribution plots and (HR)TEM images of the MMO samples. (**a**) Pore size distributions plots of the samples. (**b**–**f**) (HR)TEM images for the Cu_50_Mn_25_Al_25_-MMO (**b**,**c**), Cu_50_Mn_30_Al_20_-MMO (**d**), Cu_50_Zn_25_Al_25_-MMO (**e**), and Cu_50_Mg_25_Al_25_-MMO (**f**) samples. The inserted pore size distribution plot of Figure 2b is obtained by counting the sizes of 300 pores in the TEM image. Corresponding N_2_ adsorption/desorption isotherm and pore size distributions in the range of 0–70 nm for the samples are shown in Figure S4.

Figure 2 displays pore size distribution plots and (HR)TEM images of the samples. Interestingly, the Cu_50_Mn_25_Al_25_-MMO and Cu_50_Mn_30_Al_20_-MMO samples have a large number of 3–6 nm pores, which are much more than that of the Cu_50_Zn_25_Al_25_-MMO and Cu_50_Mg_25_Al_25_-MMO samples (Figure 2a). Furthermore, the (HR)TEM images also demonstrate the enrichment pores with the size of ~5 nm in the Cu_50_Mn_25_Al_25_-MMO sample (Figure 2b,c and Figure S3). These HRTEM images show that these pores should originate from the stacking of the CuO and MnAl_2_O_4_ nanoparticles (as marked by the red cycle in Figure 2c). The other Cu-Mn-Al MMO samples also contain similar pores as shown in Figure S4. By comparison, there are only a few similar pores in the Cu_50_Zn_25_Al_25_-MMO and Cu_50_Mg_25_Al_25_-MMO samples (Figure 2a,e,f and Figure S3). Additionally, the CuMn catalyst is constructed by non-ordered stacking of CuO and CuMn_2_O_4_ nanoparticles (Figures S2 and S5). The pore sizes of CuMn catalyst are larger than 3.6 nm and distributed in a wide range (Figure S4). Additionally, the *S*_BET_ of CuMn catalyst is much smaller than all the MMO samples (Table 1). These properties may be closely correlated with the large sizes and non-ordered stacking of the nanoparticles (Figures S4 and S5). Therefore, the MMO samples derived from Cu-Mn-Al LDH possess abundant pores of 3–6 nm and high surface areas, which will provide plentiful active sites and therefore be beneficial for the catalytic reaction.

**Figure 3:**
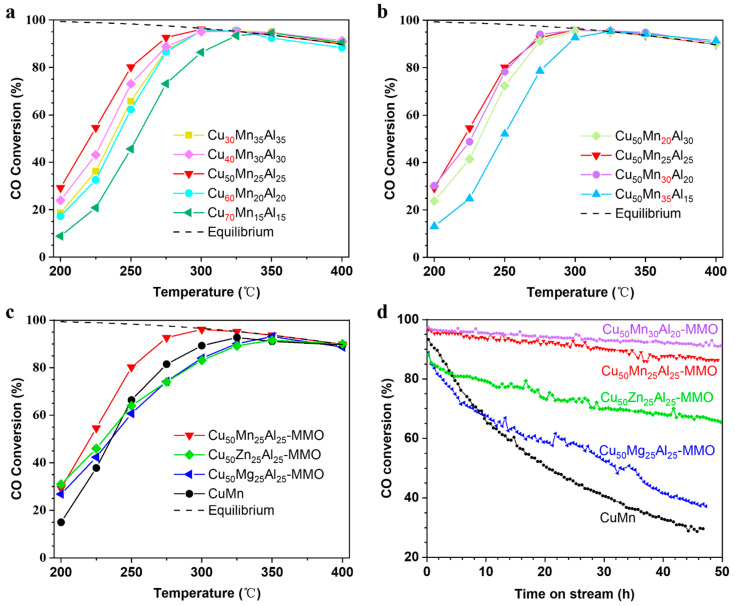
Catalytic performance of the MMO samples after activation. (**a**–**c**) Temperature-dependent activities of the catalysts. (**d**) Long-term stability test of the WGS reaction at 300 °C. Reaction conditions: CO/H_2_/CO_2_/N_2_ = 14.9/27.2/7.3/50.6 (molar ratio), GHSV = 24,000 ${mL\cdot g}_{cat}^{-1}$·h^−1^ (steam not included), H_2_O/CO = 4/1 (molar ratio), 50 mg catalyst. The dotted line indicates the CO thermodynamic equilibrium conversion that was calculated according to the reported method [41].

Figure 3 demonstrates the catalytic performance of the MMO samples for the WGS reaction. For the MMO catalysts at a fixed Mn/Al ratio of 1/1, CO conversions increased between 200 °C and 275 °C (Figure 3a). It is noted that the CO conversion increased with the increasing of Cu content from 30 to 50% and reduced as the Cu content increased from 50 to 70% (Figure 3a). At reaction temperature above 300 °C, CO conversions were very close for all the catalysts due to the achievement of equilibrium conversion and showed a downward trend with the increasing reaction temperature. For the supported catalysts, maximizing the exposed surface of metal particles is usually beneficial to enhancing the catalytic activity [35,36]. However, for the WGS reaction, there is general agreement that maximizing the density of the metal/support interfaces increases the catalytic activity [14,18,37,38]. Evidently, too high or too low Cu content in the Cu-Mn-Al MMO is not conducive to maximizing the interface between the Cu particles and Mn or/and Al oxides. Regardless of the influence of dispersion state and particle sizes, it should be helpful to maximize the metal–oxide interface when the catalyst has similar content of metal and oxides. The experimental results display that the Cu_50_Mn_25_Al_25_-MMO catalyst with Cu content of ~50 at.% exhibited a high reaction rate of 42.6 ${\mu \mathrm{mol}}_{\mathrm{CO}}^{-1}\cdot g_{\mathrm{cat}}^{-1}\cdot s^{-1}$ (with CO conversion of 96.1%), which located it at the top level of non-precious metal catalysts (Table S1).

For the MMO catalysts at a fixed Cu content of 50%, CO conversions of the Cu_50_Mn_25_Al_25_-MMO and Cu_50_Mn_30_Al_20_-MMO catalysts below 300 °C were close to each other but higher than that of the Cu_50_Mn_20_Al_30_-MMO and Cu_50_Mn_35_Al_15_-MMO catalysts (Figure 3b). It indicated that appropriate Mn content is needed for high activity in the WGS reaction. For the Cu_50_Mg_25_Al_25_-MMO and Cu_50_Zn_25_Al_25_-MMO catalysts, the CO conversions were initially at a high level (at 200 °C) and increased slowly as the temperature rose from 200 to 300 °C (Figure 3c). Contrarily, CO conversion of the CuMn catalyst increased quickly with the rising temperature (Figure 3c). CO conversion of the Cu_50_Mn_25_Al_25_-MMO catalyst was at a high level initially and increased quickly as the temperature rose from 200 to 300 °C (Figure 3b). These results indicate that the Mn components present considerable effects on the catalytic activity of WGS reaction.

The Mn-containing MMO catalysts (the Cu_50_Mn_25_Al_25_-MMO and Cu_50_Mn_30_Al_20_-MMO samples) exhibited impressive catalytic activities in the WGS reaction as described above. Significantly, the Mn-containing MMO catalysts also delivered outstanding catalytic stability, as shown in Figure 3d. After 50 h on-stream measurement, the catalytic activity decreased slightly for the Cu_50_Mn_30_Al_20_-MMO. The Cu_50_Mn_25_Al_25_-MMO catalyst presented lower catalytic stability than the Cu_50_Mn_30_Al_20_-MMO catalyst, but it was still much more stable than the Cu_50_Zn_25_Al_25_-MMO, Cu_50_Mg_25_Al_25_-MMO, and CuMn catalysts (Figure 3d). The CuMn catalyst showed a high activity initially but decreased quickly over time, which is much different from the cases of Mn-containing MMO catalysts. As it was known, the aluminum component was a typical structural promoter, which can increase stability by preventing catalyst sintering [39,40]. It could explain why there was such a significant difference in the catalytic stability between the Cu-Mn-Al MMO and CuMn catalysts. However, it cannot elucidate the poor stability of the Cu_50_Zn_25_Al_25_-MMO and Cu_50_Mg_25_Al_25_-MMO catalysts. This contradiction inspires us to obtain insight into the underlining mechanism.

**Figure 4:**
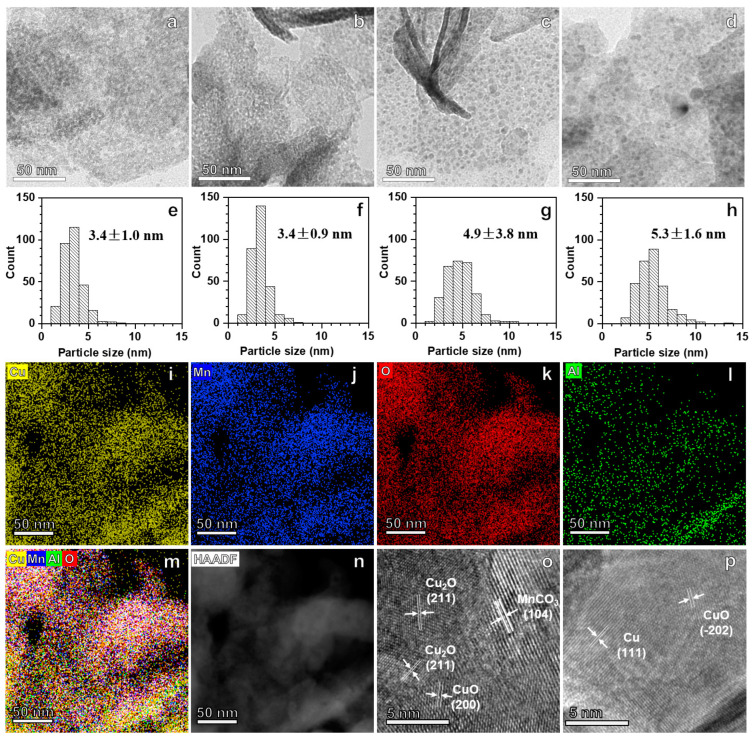
Microscopic characterizations of the catalysts after 5 h on stream in WGS reaction. (**a**–**d**) TEM images of the spent catalysts. (**e**,**f**) The particle sizes distribution of the spent catalysts. (**i**–**n**) The EDX mapping and corresponding HAADF-TEM image of the spent Cu_50_Mn_25_Al_25_-MMO catalyst. (**o**,**p**) HRTEM images of the spent catalysts. (**a**,**e**,**o**) Cu_50_Mn_25_Al_25_-MMO, (**b**,**f**) Cu_50_Mn_30_Al_20_-MMO, (**c**,**g**,**p**) Cu_50_Zn_25_Al_25_-MMO, (**d**,**h**) Cu_50_Mg_25_Al_25_-MMO.

Figure 4 exhibits the microscopic structure and morphology of the spent catalysts in the WGS reaction for 5 h. The average particle sizes of the Cu_50_Mn_25_Al_25_-MMO and Cu_50_Mn_30_Al_20_-MMO catalysts (Figure 4a,b,e,f) were only 3.4 nm, while they were 4.9, 5.3 and 12.7 nm for the Cu_50_Zn_25_Al_25_-MMO (Figure 4c,g), Cu_50_Mg_25_Al_25_-MMO (Figure 4d,h) and CuMn (Figure S6) catalysts, respectively. In addition to the smaller average sizes, the particle sizes of the Cu_50_Mn_25_Al_25_-MMO and Cu_50_Mn_30_Al_20_-MMO catalysts were distributed in a very narrow range compared to the other catalysts (Figure 4e–h and Figure S6). The EDX mapping and corresponding HAADF-TEM image demonstrated that the Cu, Mn and Al components were well dispersed in the spent Cu_50_Mn_25_Al_25_-MMO catalyst (Figure 4i–n). Benefitting from the smaller sizes of particles and the well dispersion of metal components, the density of the metal/support interfaces for the Cu-Mn-Al catalysts sharply increased, and, therefore, the catalytic activities should have been intensively improved [14,18,37,38]. In other words, the high activity of the CuMnAl-MMO catalysts could be attributed to the rich interfaces between Cu-containing particles and manganese oxide particles. In addition, the XRD patterns and HRTEM images (Figure 4o,p and Figure S7) showed that there are Cu, Cu_2_O, and CuO phases in the spent catalysts. Interestingly, it could find such a rule that the catalyst with better catalytic stability contained less Cu and more Cu_2_O (Figure 3d and Figure S7). Further studies are needed to reveal the mechanism of this rule.

**Figure 5:**
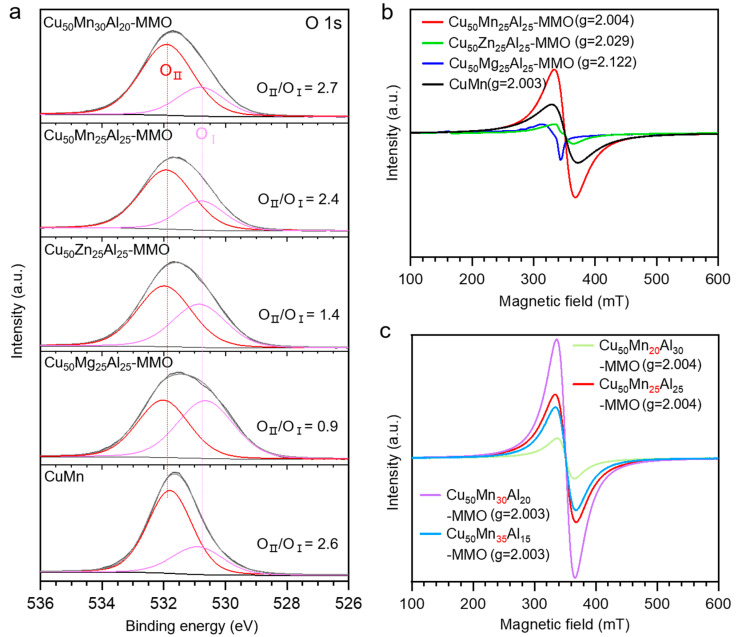
O 1s XPS spectra (**a**) and EPR spectra (**b**,**c**) for the catalysts after 5 h on stream in WGS reaction. The reaction conditions are the same as those of the long-term stability test (Figure 3d).

Figure 5 is the XPS spectra and EPR spectra for the spent catalysts. The O1s XPS spectra were deconvolved into two fitted peaks O_I_ and O_II_ (Figure 5a), representing two different kinds of oxygen species. The peak O_I_ at 530.7 ± 0.15 eV corresponded to the lattice oxygen bound to metal cations [30,31,42]. The peak O_II_ at 531.9 ± 0.1 eV was mainly assigned to the adsorbed surface oxygen on oxygen vacancies, including the surface hydroxyl-like species [30,31,42]. The O_II_/O_I_ ratio could qualitatively estimate the ratio of surface oxygen to lattice oxygen, thus, the higher O_II_/O_I_ ratio usually suggests the more oxygen vacancies [26,29,30]. Creating an oxygen-vacancy-rich surface may be an effective approach to enhance the activation of H_2_O, and, therefore, may improve the catalytic performance of the WGS reaction [26,43]. The Mn-containing catalysts, i.e., Cu_50_Mn_30_Al_20_-MMO, Cu_50_Mn_25_Al_25_-MMO, and CuMn, all have high O_II_/O_I_ ratios above 2.4, which are much higher than the Cu_50_Zn_25_Al_25_-MMO and Cu_50_Mg_25_Al_25_-MMO catalysts. The catalysts with high O_II_/O_I_ ratios display high catalytic activities for WGS reactions between 250 and 300 °C (Figure 3b,c and Figure 5a), suggesting the important role of oxygen vacancies on the catalytic activity of the catalysts.

EPR results exhibit that the Mn-containing catalysts all have a g value of 2.003 or 2.004 (Figure 5b,c), which proves the existence of oxygen vacancies [44]. For the Cu-Mn-Al MMO catalysts, the intensity of EPR spectra increases with the rise of Mn content except the Cu_50_Mg_35_Al_15_-MMO catalyst (Figure 5c). It suggests that appropriate content of Mn may be beneficial to improving the amount of oxygen vacancies for the Cu-Mn-Al MMO catalysts. The Cu_50_Mg_30_Al_20_-MMO and Cu_50_Mn_25_Al_25_-MMO catalysts with relatively high intensity of EPR spectra not only present high catalytic activity in WGS, but also deliver outstanding catalytic stability (Figure 3d).

**Figure 6:**
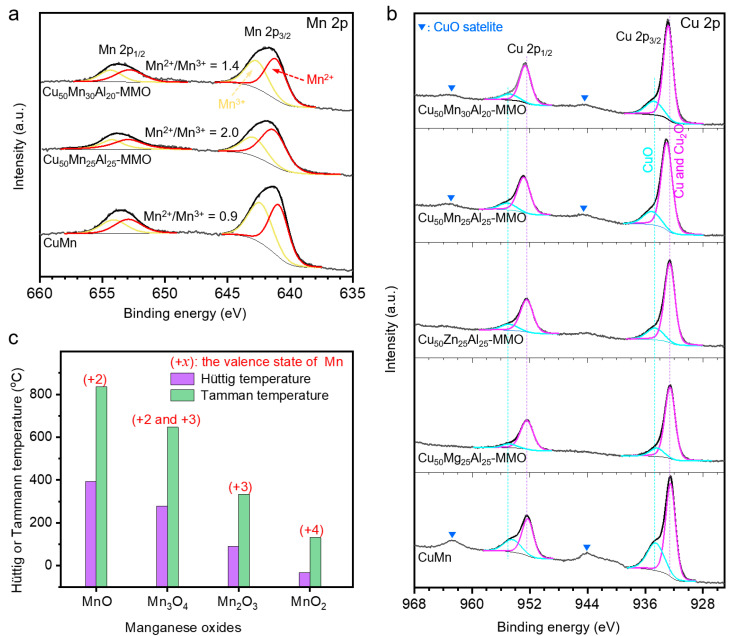
XPS spectra for the spent catalysts and physical/chemical properties for manganese oxides. (**a**,**b**) Mn 2p (**a**) and Cu 2p (**b**) XPS spectra of the catalysts after 5 h on stream in WGS reaction. The reaction conditions are the same as those of the long-term stability test (Figure 3d). (**c**) The Mn valence state, Hüttig temperature and Tamman temperature for manganese oxides, and more details are shown in Table S2.

Figure 6 shows the XPS spectra of the spent catalysts and the relationship between the Hüttig/Tamman temperature and the Mn valence state for manganese oxides. Mn 2p XPS spectra show that the Mn components in the catalysts presented as divalent (Mn^2+^) and trivalent (Mn^3+^) forms (Figure 6a). The ratio of Mn^2+^/Mn^3+^ is 0.9, 2.0, and 1.4 for the CuMn, Cu_50_Mg_25_Al_25_-MMO, and Cu_50_Mg_30_Al_20_-MMO catalysts, respectively. It suggests that Mn components in the Cu-Mg-Al MMO catalysts present a relatively low-valence state. Rhodochrosite (MnCO_3_) was the only Mn phase detected by the XRD measurement in the spent catalysts (Figure S7), but it easily decomposes into MnO at temperatures above 200 °C. Consequently, MnO should be the main Mn-containing phase for the Cu-Mg-Al MMO catalysts in the WGS operation condition at temperatures around 300 °C. By comparison, Mn_2_O_3_ and/or Mn_3_O_4_ may be the main Mn-containing phase for the CuMn catalyst. Figure 6b presents the Cu 2p XPS spectra of spent catalysts. There are two fitting peaks at 932.7 ± 0.2 eV and 934.8 ± 0.3 eV in the Cu 2p_3/2_ photoelectron peaks, which could be assigned to Cu/Cu_2_O and CuO, respectively. According to the area of the fitting peaks, the Cu and/or Cu_2_O are the main phases for Cu components in the spent catalysts. Since the binding energy of Cu 2p for pure Cu and pure Cu_2_O are very close (at about 932.5–932.7 eV) [45,46,47], it is hard to separate the peaks of Cu and Cu_2_O from each other. Combined with the XRD patterns (Figure S7), it can be deduced that Cu_2_O should be the main Cu-containing phase for the spent Cu_50_Mn_30_Al_20_-MMO and Cu_50_Mn_25_Al_25_-MMO catalysts.

Catalyst sintering is considered as the primary reason for the deactivation of WGS catalysts besides sulfur poisoning [48,49]. The rate of catalyst sintering has a strong correlation with operation temperature [50,51]. The Hüttig temperature (*T*_Hüt_) and Tamman temperature (*T*_Tam_) can roughly estimate the sinter temperature for different materials [50]. The atoms at the particle surface start to exhibit mobility at *T*_Hüt_, and the atoms from bulk start to mobile above the *T*_Tam_ [50]. As shown in Figure 6b, the *T*_Hüt_ and *T*_Tam_ of the manganese oxides sharply decreased with the increase in the valence state of manganese. It means that the Mn-containing particles in a high valence state are mobile and aggregate into larger particles. In contrast, the Mn-containing particles in a low valence state would have good anti-sintering performance. In fact, the Cu_50_Mn_30_Al_20_-MMO and Cu_50_Mn_25_Al_25_-MMO catalysts with a low-valence state of manganese exhibit much higher catalytic stability than the CuMn catalyst, as mentioned above.

Since the *T*_Hüt_ is as high as 392 °C, the surface atoms of MnO cannot migrate below this temperature; this is because the oxygen vacancies (Mn^2+^-O_v_-Mn^2+^) should be very stable below this temperature, which is beneficial to improving the catalytic durability of the catalysts besides the activation of H_2_O. Recently, Xi and his co-workers [26] have also reported that stabilizing the oxygen vacancies is a feasible pathway to enhance the durability of Pt cluster catalysts supported on reduced MoO_3_ monoliths. Based on this reason, the Cu-Mn-Al MMO catalysts of rich and stable oxygen vacancies are more durable than the Cu_50_Zn_25_Al_25_-MMO and Cu_50_Mg_25_Al_25_-MMO catalysts.

## 4. Conclusions

A series of high-purity Cu-Mn-Al LDH with high Cu content have been prepared by coprecipitation method under low supersaturation. The Cu-Mn-Al MMO derived from these LDH samples features a large number of 3–6 nm pores that are much different from the MMO without manganese component. The Cu-Mn-Al MMO catalysts exhibited outstanding catalytic activity and excellent stability in the WGS reaction. The optimized Cu_50_Mn_25_Al_25_-MMO catalyst displayed an exceptionally high reaction rate of 42.6 ${\mu \mathrm{mol}}_{\mathrm{CO}}^{-1}\cdot g_{\mathrm{cat}}^{-1}\cdot s^{-1}$, while the CO conversion was as high as 96.1% simultaneously. The outstanding catalytic activity for Cu-Mn-Al MMO catalysts should benefit from the rich interfaces between Cu-containing particles, manganese oxide particles, and/or abundant oxygen vacancies. Furthermore, the Cu-Mn-Al MMO catalysts demonstrated excellent catalytic stability during the durability test. The excellent stability may originate from the manganese oxides within a low valence state, which have good anti-sintering properties and can stabilize oxygen vacancies.

## Figures and Tables

**Table 1:** Physicochemical properties of different MMO samples.

Samples	Designed Contents ^a^ (Cu:M:Al ^b^)	Detected Contents (at.%) ^c^					*S*_BET_ (m^2^·g^−1^)
		Cu	Mn	Al	Zn	Mg	
Cu_30_Mn_35_Al_35_-MMO	30:35:35	29.9	34.6	35.5	-	-	122
Cu_40_Mn_30_Al_30_-MMO	40:30:30	40.2	29.8	30.0	-	-	117
Cu_50_Mn_25_Al_25_-MMO	50:25:25	50.6	25.6	23.8	-	-	88
Cu_60_Mn_20_Al_20_-MMO	60:20:20	60.8	19.7	19.5	-	-	74
Cu_70_Mn_15_Al_15_-MMO	70:15:15	70.4	15.0	14.7	-	-	71
Cu_50_Mn_20_Al_30_-MMO	50:20:30	50.7	19.3	30.1	-	-	72
Cu_50_Mn_30_Al_20_-MMO	50:30:20	50.8	29.2	20.0	-	-	73
Cu_50_Mn_35_Al_15_-MMO	50:35:15	50.9	34.4	14.7	-	-	62
Cu_50_Zn_25_Al_25_-MMO	50:25:25	49.3	-	25.3	25.4	-	67
Cu_50_Mg_25_Al_25_-MMO	50:25:25	50.9	-	26.2	-	22.8	58
CuMn	50:50:0	50.8	49.2	-	-	-	37

Note: ^a^ Designed contents represent the designed molar percentage of corresponding metals in the preparation process. ^b^ M represents the metal of Mn, Zn or Mg. ^c^ The detected contents of metals for different samples were measured by ICP-OES.

## Data Availability

The data presented in this study are available on request from the corresponding author.

## Supplementary Materials

The following supporting information can be downloaded at: https://www.mdpi.com/article/10.3390/molecules28041522/s1, Figure S1: XRD patterns of the mixed metal oxides (MMO) derived from layered double hydroxides (LDH) samples; Figure S2: XRD pattern of the CuMn sample; Figure S3: HRTEM images of the MMO samples; Figure S4: N_2_ adsorption/desorption isotherms (a) and pore size distributions (b–d) of the samples; Figure S5: TEM image and particle size distribution for the CuMn sample; Table S1: Comparison of the activities of the representative catalytic systems with non-noble metal for the WGS reaction; Figure S6: TEM image and particle size distribution for the CuMn catalyst after 5 h on stream in WGS reaction; Figure S7: XRD pattern of the spent catalysts in WGS reaction for 5 h; Table S2: The melting point, Hüttig temperature and Tamman temperature of manganese oxides [52,53,54,55,56].

## Sample Availability

Samples of the catalysts are available from the authors.
