# Supplementary material for: Interfaces and Oxygen Vacancies-Enriched Catalysts Derived from Cu-Mn-Al Hydrotalcite towards High-Efficient Water–Gas Shift Reaction
Source: Molecules. 2023 Feb 4;28(4):1522. doi: 10.3390/molecules28041522 (PMC9966559; doi:10.3390/molecules28041522)
Supplement: Supplementary file 1 [file molecules-28-01522-s001.zip › molecules-2185446-supplementary.pdf]

Supplementary Materials for

Interfaces and Oxygen Vacancies-Enriched Catalysts Derived from Cu-Mn-Al Hydrotalcite towards High-Efficient Water-Gas Shift Reaction

Hanci Li[1], Zhenyi Xiao[1], Pei Liu[1], Hairu Wang[1], Jiajun Geng[1], Huibin Lei[1, 2], Ou Zhuo[1, 2, *]

[1] College of Chemistry and Chemical Engineering, Jishou University, Jishou 416000, China

[2] Hunan Province Key Laboratory of Mineral Cleaner Production and Green Functional Materials, Jishou University, Jishou 416000, China

[*] Correspondence: zhuoouhao@126.com (O.Z.)

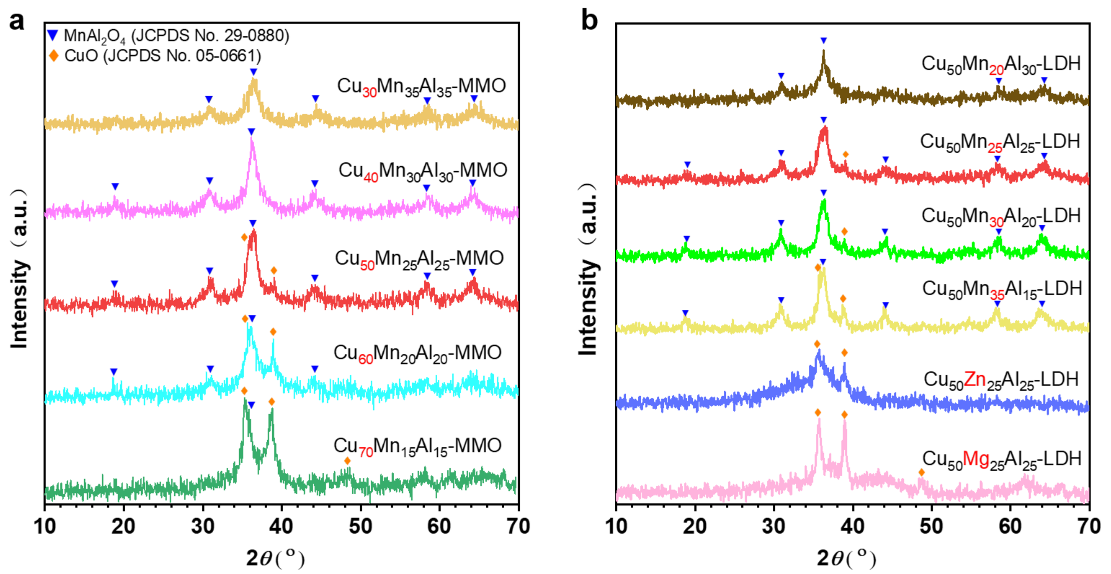

Figure S1. XRD patterns of the mixed metal oxides (MMO) derived from layered double hydroxides (LDH) samples.

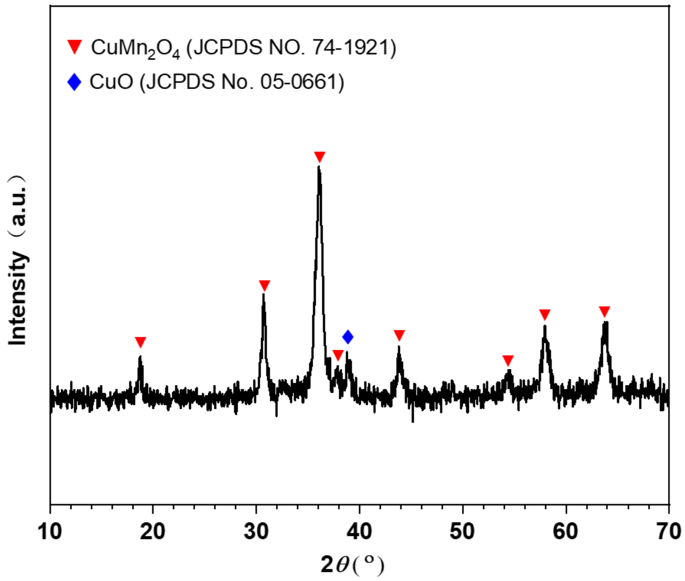

Figure S2. XRD pattern of the CuMn sample (calcined at 500°C for 3 h).

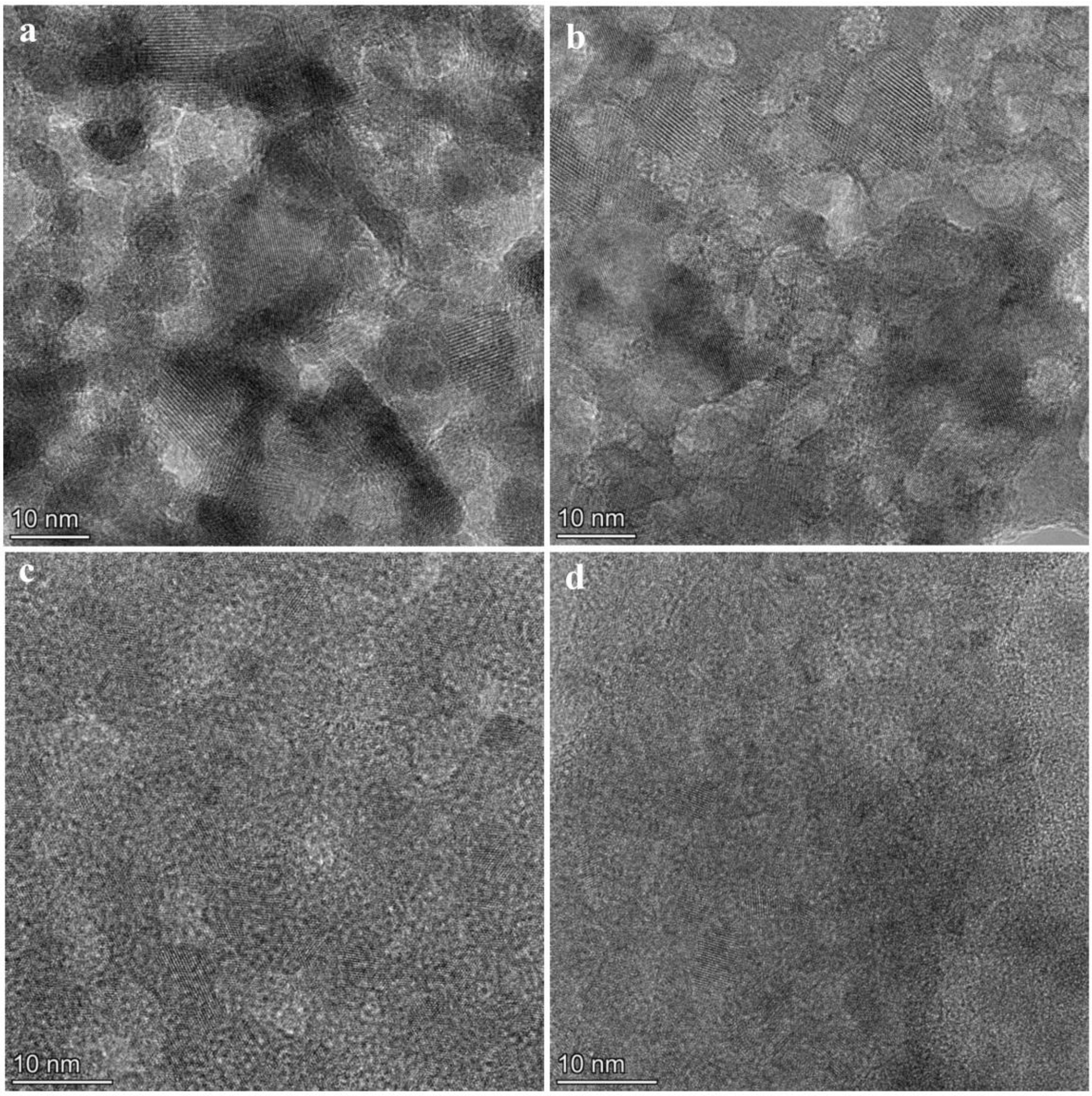

Figure S3. HRTEM images of the MMO samples. (a, b) $Cu_{50}Mn_{25}Al_{25}$-MMO, (c) $Cu_{50}Zn_{25}Al_{25}$-MMO, (d) $Cu_{50}Mg_{25}Al_{25}$-MMO.

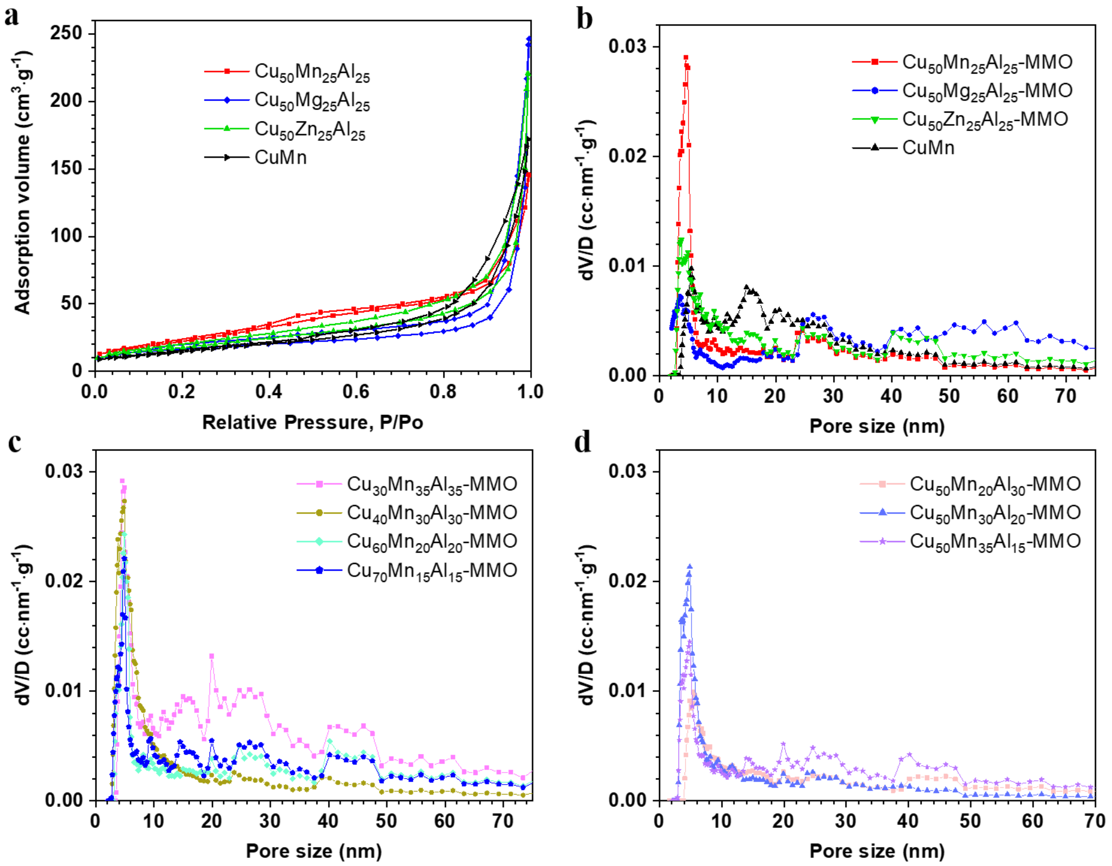

Figure S4. N_2 adsorption/desorption isotherms (a) and pore size distributions (b-d) of the samples.

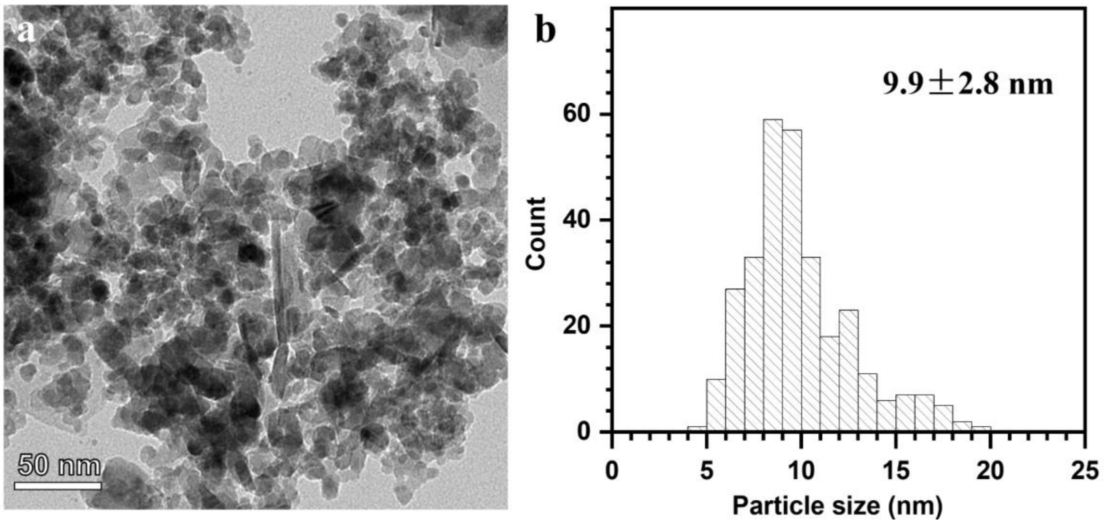

Figure S5. TEM image and particle size distribution for the CuMn sample.

Table S1. Comparison of the activities of the representative catalytic systems with non-noble metal for the WGS reaction

Catalysts	*T* (K)	Gas feed ($CO : H_2 : CO_2$)	CO/H_2O ratio	WHSV ($mL \cdot g_{cat}^{-1} \cdot h^{-1}$)	**CO conversion (%)**	**Reaction rate** [a] ($\mu mol_{CO}^{-1} \cdot g_{cat}^{-1} \cdot s^{-1}$)	Ref.
$Cu_{50}Mn_{25}Al_{25}$-MMO	473	14.9%:27.2%:7.3%	1 : 4	24000	**29.2**	**12.9**	This work
	573	14.9%:27.2%:7.3%	1 : 4	24000	**96.1**	**42.6**	
$Cu_{50}Mn_{30}Al_{20}$-MMO	473	14.9%:27.2%:7.3%	1 : 4	24000	**30.3**	**13.4**	
	573	14.9%:27.2%:7.3%	1 : 4	24000	**95.8**	**42.5**	
Commercial $Cu/ZnO/Al_2O_3$	550	5% : 0 : 0	1 : 2	36000	**50**	**12**	Zhang, Z., et al. *Nature Communications*, 2017, 8, 488.
$20Cu5Fe/SiO_2$	603	2.5%: 0 : 0	1 : 1	-	**-**	**~3.6**	Zhu M., et al. *Angew. Chem. Int. Ed.*, 2019, 58, 9083.
Cu-Mg_HAl_H	523	19%: 0 : 0	1 : 3	5520	**97.3**	**12.7**	Lee C. H., et al. *Renewable and Sustainable Energy Reviews*, 2021, 145, 111064.
$Cu_{0.3}Fe_{0.7}O_x$	473	2.2%: 0 : 0	1 : 7	42000	**~73**	**~8.3**	Yan H., et al. *Applied Catalysis B: Environmental*, 2018, 226, 182.
MgCuCe catalyst	573	6.3%:37.5%:6.3%	1 : 6	120000	**~66**	**83**	Jin S., et al. *Applied Catalysis B: Environmental*, 2021, 284, 119701.
Inverse CeO_2/Cu catalyst	573	2% : 0 : 0	1 : 5	-	**-**	**47.3**	Yan, H., et al. *Nature Communications*, 2019, 10, 3470.

T is the reaction temperature. [a] Micromoles of CO per grams of catalyst per second.

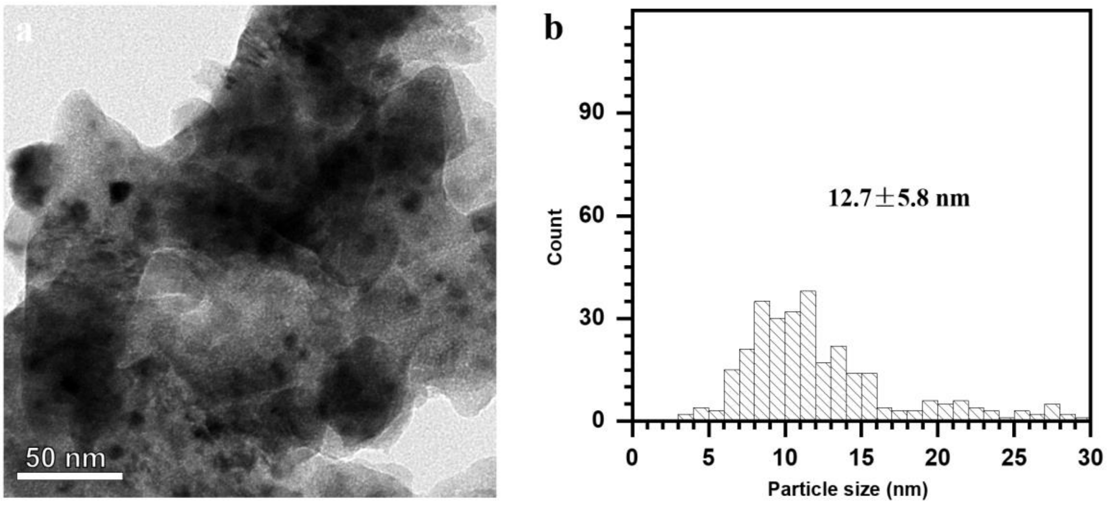

Figure S6. TEM image and particle size distribution for the CuMn catalyst after 5 h on stream in WGS reaction.

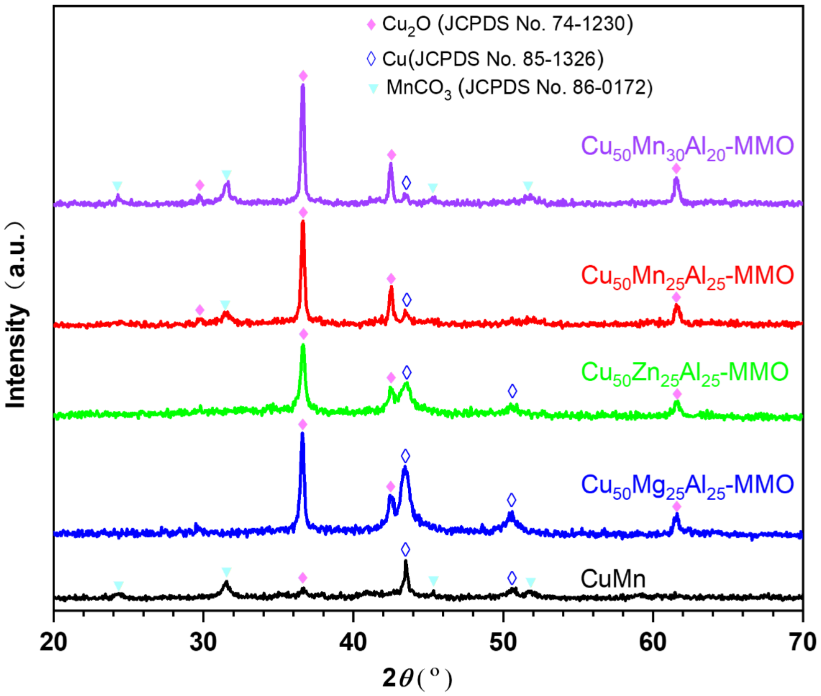

Figure S7. XRD pattern of the spent catalysts in WGS reaction for 5 h.

Table S2. The melting point, Hüttig temperature and Tamman temperature of manganese oxides [a].

Oxides	Melting point [b] (T_{melt})	Hüttig temperature ($T_{Hüt}$)	Tamman temperature (T_{Tam})
MnO	2218 K (1945 ^oC)	665 K (392 ^oC)	1109 K (836 ^oC)
Mn_3O_4	1840 K (1567 ^oC)	552 K (279 ^oC)	920 K (647 ^oC)
M_2O_3	1213 K (940 ^oC)	364 K (91 ^oC)	607 K (334 ^oC)
MnO_2	808 K (535 ^oC)	242 K (-31 ^oC)	404 K (131 ^oC)
$MnCO_3$	- (decompose)	-	-
Cu	1356 K (1083 ^oC)	407 K (134 ^oC)	678 K (405 ^oC)
Cu_2O	1508 K (1235 ^oC)	452 K (179 ^oC)	754 K (481 ^oC)
CuO	1719 K (1446 °C)	516 K (243 ^oC)	860 K (587 ^oC)

[a] The Hüttig temperature and Tamman temperature are calculated from the corresponding melting point using the following equations: $T_{Hüt} = 0.3T_{melt}$, $T_{Tam} = 0.5T_{melt}$. The temperature used in the calculation process is absolute temperature. [b] The melting point is obtained from the following literatures: Navasery M., et al. *Mordern Physics Letters B*, 2012, 26(6), 1150039; Moiseev G. K., et al. *Inorganic Materials*, 2006, 42, 632; Kosenko A. V., et al. *Journal of Phase Equilibria*, 2001, 22, 12.
